# A *Wolbachia w*Mel Transinfection in *Aedes albopictus* Is Not Detrimental to Host Fitness and Inhibits Chikungunya Virus

**DOI:** 10.1371/journal.pntd.0002152

**Published:** 2013-03-28

**Authors:** Marcus S. C. Blagrove, Camilo Arias-Goeta, Cristina Di Genua, Anna-Bella Failloux, Steven P. Sinkins

**Affiliations:** 1 University of Oxford, Peter Medawar Building for Pathogen Research and Departments of Medicine (NDM)/Zoology, Oxford, United Kingdom; 2 Institut Pasteur, Department of Virology, Paris, France; Monash University, Australia

## Abstract

**Background:**

*Wolbachia* inherited intracellular bacteria can manipulate the reproduction of their insect hosts through cytoplasmic incompatibility (CI), and certain strains have also been shown to inhibit the replication or dissemination of viruses. *Wolbachia* strains also vary in their relative fitness effects on their hosts and this is a particularly important consideration with respect to the potential of newly created transinfections for use in disease control.

**Methodology/Principal Findings:**

In *Aedes albopictus* mosquitoes transinfected with the *w*Mel strain from *Drosophila melanogaster*, which we previously reported to be unable to transmit dengue in lab challenges, no significant detrimental effects were observed on egg hatch rate, fecundity, adult longevity or male mating competitiveness. All these parameters influence the population dynamics of *Wolbachia*, and the data presented are favourable with respect to the aim of taking *w*Mel to high population frequency. Challenge with the chikungunya (CHIKV) virus, for which *Ae. albopictus* is an important vector, was conducted and the presence of *w*Mel abolished CHIKV dissemination to the saliva.

**Conclusions/significance:**

Taken together, these data suggest that introducing *w*Mel into natural *Ae. albopictus* populations using bidirectional CI could be an efficient strategy for preventing or reducing the transmission of arboviruses by this species.

## Author Summary

The tiger mosquito *Aedes albopictus* is an invasive disease vector whose range has expanded throughout the tropics, and some temperate regions, in recent decades from its native South East Asia. It is an important vector of human viruses including dengue and chikungunya; in recent years a mutation has been detected in chikungunya virus that specifically increases transmission efficiency by *Ae. albopictus*, causing concern that epidemics of this disease will become more widespread and severe. Here we show that when transinfected with a strain of the symbiont *Wolbachia* called *w*Mel, originating in fruitflies, the ability of the mosquito to transmit the mutated chikungunya virus was abolished in lab experiments. Furthermore, the *w*Mel strain was shown to produce no detectable fitness costs in this new host, examining numbers of eggs produced, egg hatch, lifespan, and male mating competitiveness compared to wildtypes. This is encouraging with respect to developing the system for use in the control of dengue and chikungunya viruses.

## Introduction

*Aedes albopictus* is a medically important invasive mosquito that has expanded from Asia into many new regions of the tropics and also into temperate regions of the Americas and Europe; it transmits a number of arboviruses including dengue and chikungunya [1]. All known wild populations of *Ae. albopictus* are naturally superinfected at very high frequency with two strains of the maternally inherited bacterium *Wolbachia pipientis*, called *w*AlbA and *w*AlbB [2], [3]. Recently, we reported the generation of an *Ae. albopictus* line transinfected with the *w*Mel strain of *Wolbachia* from *Drosophila melanogaster*, which proved unable to transmit dengue virus in lab challenges and induced complete bidirectional CI when crossed with a naturally *w*AlbA plus *w*AlbB (*w*AlbA/B)-infected line [4]. The *w*Mel strain, and the related *w*MelPop [5] have also been shown to strongly inhibit viral replication in *Aedes aegypti*, as well as causing chronic immune upregulation in that naturally *Wolbachia*-uninfected host [6]–[8].

If *w*Mel is to be used to reduce the dengue transmission capacity of wild *Ae. albopictus*, a critical factor is whether mosquitoes infected with this strain incur major fitness penalties. It has previously been reported that the natural *w*AlbA/B strain superinfection actually provides a fitness advantage in the form of increased longevity and fecundity compared to an uninfected line [9], [10]; given that *w*Mel is not specifically adapted to *Ae. albopictus* and *vice versa*, significant fitness costs compared to wildtypes are possible. The over-replicating *w*MelPop strain variant is virulent and can approximately halve the lifespan of both its *D. melanogaster* and *Ae. aegypti* hosts [11], [12], and a *w*MelPop transinfection in *Ae. albopictus* produced a greatly reduced egg hatch from intra-strain matings [13]. Population dynamic mathematical models have emphasised the importance of fitness parameters in *Wolbachia*-based population replacement strategies [14], [15]. It is therefore very important to gain a comprehensive understanding of any fitness consequences of replacing *w*AlbA/B with *w*Mel in *Ae. albopictus*, including longevity of both male and female mosquitoes, fecundity and egg fertility rates for females and relative mating competitiveness for males.

Chikungunya virus (CHIKV) is viewed as an important potential emerging or re-emerging pathogen in many parts of the world [15], [16]. There have been a number of recent epidemics where *Ae. albopictus* has been incriminated as a major vector or as the sole vector, for example in the Indian Ocean (La Reunion), Italy, southeastern France, central Africa, and the Kerala region of India [17]–[24]. It has been of particular concern that two viral adaptations, single amino acid substitutions in the envelope glycoproteins (E1-A226V which arose around 2005 and E2-L210Q identified in 2009), have significantly improved the efficiency of dissemination and transmission by *Ae. albopictus* [25]–[28]. Therefore another major aim of this study was to assess whether the *w*Mel-transinfected line shows any impairment in its ability to transmit CHIKV, in light of the strong inhibition of dengue virus that has already been observed in this line [4], and that fact that a *w*MelPop *Wolbachia* transinfection in *Ae. aegypti* has been shown to produce an inhibitory effect with respect to CHIKV [7].

## Materials and Methods

### Mosquito strains

The Uju.wMel line [4] was generated by microinjection of *w*Mel from *D. melanogaster* into a *Wolbachia*-uninfected (cured) *Ae. albopictus* strain UjuT. Uju.wt was generated by introgression of the natural *w*AlbA/B infection of *Ae. albopictus* from the Ascoli strain (Italy) into UjuT background for four generations [4] followed by a further three here; the resulting line was >99% UjuT nuclear background and contained both *w*AlbA and *w*AlbB. All *Ae. albopictus* colonies in Oxford were maintained at 27°C and 70% relative humidity on a 12-h light/dark cycle.

### Fitness and male mating competitiveness

Females were blood fed at six days post eclosion, and aspirated into small tubes for individualized laying two days later. Eggs were matured for five days before being counted to give fecundity data and then hatched in deoxygenated water in small plastic vials. Second instar larvae were counted to give hatch rate. Uju.wMel female egg hatch was lower than for wildtype colony controls in early generations, likely as a result of variable *Wolbachia* density causing CI effects. Therefore, between G_6_ and G_12_ selection for high hatch was applied to make the *Wolbachia* density more uniform, by discarding approximately one quarter of broods which showed the lowest hatch rates.

Longevity was assessed in 30 cm×30 cm×30 cm BugDorm cages (MegaView Ltd., Taiwan) containing one hundred male and one hundred female mosquitoes (which were sexed as pupae). The mosquitoes were provided with moist cotton and supplied with sucrose *ad libitum*. A blood meal was provided at seven days, and subsequently every fourteen days; oviposition substrates were provided three days after blood feeding. Dead mosquitoes were counted and removed every four days. To assess developmental times, eggs were hatched and larvae reared at 100 larvae/litre and given 1 mg liver powder per larva per day; the number of adults eclosing per day was counted until all pupae eclosed.

Male mating competitiveness was assessed using three independent replicates of 50 male Uju.wMel : 50 male Uju.wt : 50 females (either Uju.wMel or Uju.wt), sexed at the pupal stage. Adults were allowed to emerge in Bugdorm cages and were left to mate for ten days. Females were then blood fed, and two days later were aspirated into small plastic vials for individualized laying. Eggs were hatched five days after laying; given the complete CI (zero egg hatch) observed in both directions in crosses between wildtype and *w*Mel-transinfected parents [4], the male parent was scored according to whether or not embryos hatched – hatching indicating that both contained the same *Wolbachia* strain. Spermathecae were dissected and examined under a dissecting microscope for the presence of stored sperm for all females that produced eggs with zero hatch, and data from any females without sperm were disregarded.

### Viral strain

CHIKV E1-226V was isolated from a patient on La Reunion Island in November 2005 [23]. This strain is characterized by a substitution of alanine by valine at position 226 of the E1 envelop glycoprotein in a region which is responsible for fusion of viral and cellular membranes within the endosome [29]. This anonymized strain was provided by the French National Reference Center for Arboviruses at the Institut Pasteur. Viral stocks were produced on *Ae. albopictus* C6/36 cells that were infected at a multiplicity of infection (MOI) of 0.1 PFU (plaque forming units)/cell for 48 h at 27°C. Stocks were constituted after two passages on C6/36 cells, titrated on Vero cells, and stored at −80°C until use.

### Mosquito infections

Mosquito adults were starved for 24 hours and then allowed to feed on artificial blood-meals consisting of a virus suspension (1∶3), washed rabbit erythrocytes (2∶3), and 5 mM ATP. The blood-meal was provided in glass feeders covered with a pig intestine membrane and maintained at 37°C. The titer of the blood-meal was 10^7.5^ PFU/mL. Females placed in plastic boxes were allowed to feed for 15 min. Engorged females were sorted on ice, transferred in cardboard containers, provided with sugar solution and maintained in BSL-3 insectaries at 28°C until day 7 post-infection (in line with previous experiments showing that viral titres gradually decreased after day 7 post infection [30])..

To evaluate transmission capacity, 35–50 females were anaesthetized on ice, their wings and legs were removed to induce stress and force salivation. The proboscis was inserted into a 20 µL tip containing 10 µL of foetal bovine serum. After 45 minutes, saliva was collected with the medium and expelled under pressure into a 0.5 mL tube containing 40 µL of DMEM. Samples were later titrated by focus fluorescent assay [31] on *Ae. albopictus* C6/36 cells. The transmission rate was estimated as the percentage of mosquitoes with infectious saliva among tested mosquitoes.

Saliva samples were titrated by focus fluorescent assay on C6/36 *Aedes albopictus* cell culture. Cells were grown to confluent monolayers in 96-well plates and infected with 10-fold serial dilutions of virus. After incubation at 28°C for 3 days, plates were stained using hyper-immune ascetic fluid specific to CHIKV (provided by the French National Reference Center of Arboviruses at the Institut Pasteur) as the primary antibody and a conjugate anti-IgG mouse (Bio-Rad) as the secondary antibody. The focus fluorescent units were counted with a fluorescent microscope and the titer was calculated as FFU/saliva.

## Results

### Uju.wMel fitness parameters and male mating competitiveness

**Figure 1:**
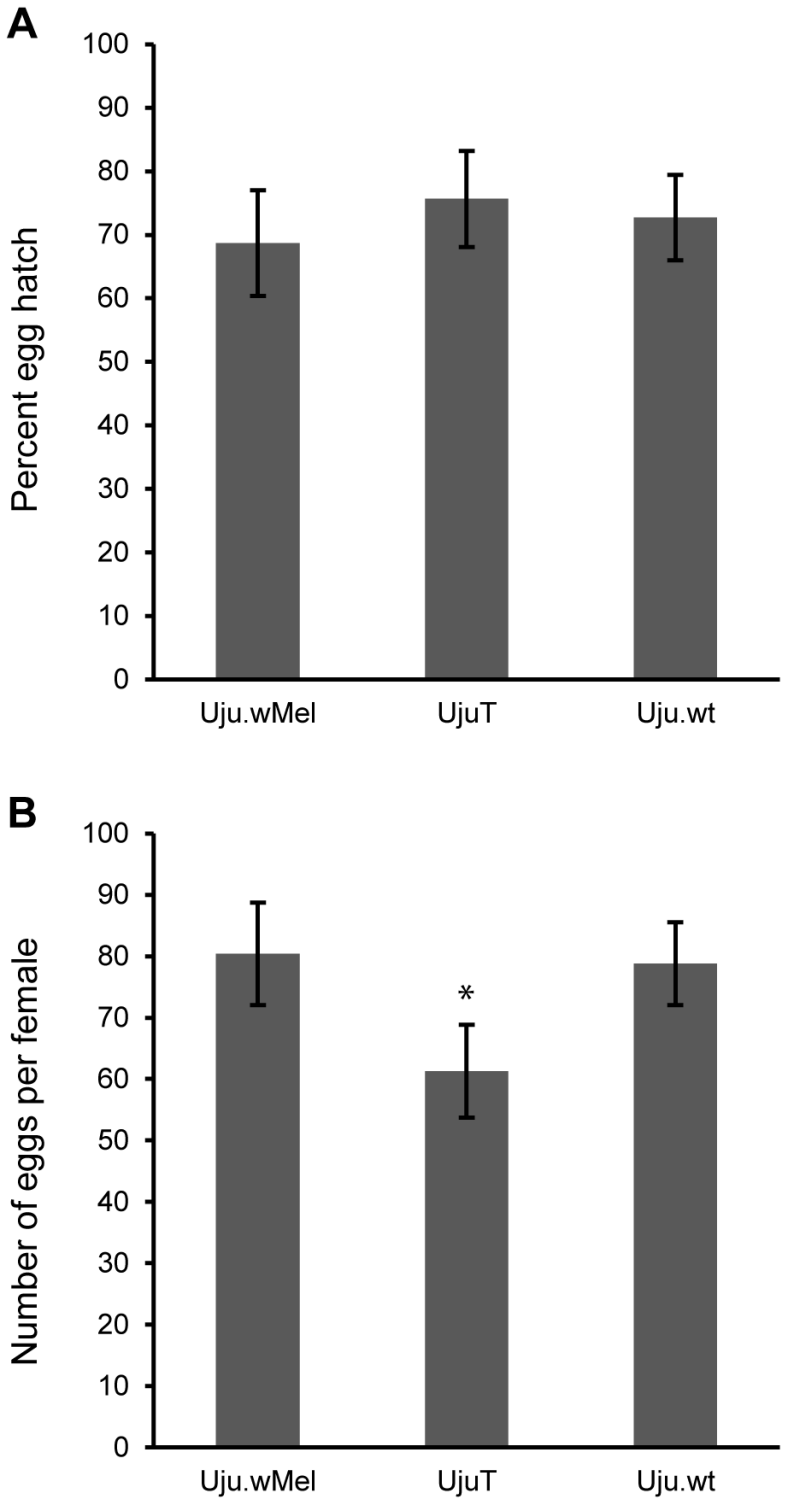
Hatch rate and fecundity of Uju.wMel. Egg hatch (A) and fecundity or mean number of eggs produced per female per gonotrophic cycle (B) of Uju.wMel was assessed at generation sixteen. Females were blood fed at six days post eclosion, individualized for laying, and eggs hatched after five days. Second instar larvae were counted to calculate percent hatch (A) and eggs per batch per female counted to give fecundity (B). A: Uju.wMel n = 452, UjuT n = 858, Uju.wt n = 508. B: Uju.wMel n = 16, UjuT n = 14, Uju.wt n = 20. Error bars represent the SEM.

The egg hatch rate and the fecundity per single gonotrophic cycle observed in the *w*Mel-transinfected *Ae. albopictus* line Uju.wMel [4] were not significantly different to the natural *w*AlbA/B-superinfected strain Uju.wt (hatch rate, *p* = 0.369, Wilcoxon rank sum test; fecundity, *p* = 0.738, Wilcoxon rank sum test) (figure 1A & B). This is consistent with observations in other novel transinfections over time [32]–[34]. Additionally, the fecundity of Uju.wt was significantly higher than UjuT (*p* = 0.028, Wilcoxon rank sum test), supporting previous reports of increased fecundity in the presence of the natural *Ae. albopictus Wolbachia* superinfection compared to a cured *Wolbachia*-uninfected line [9], [10]. As with all characterization experiments presented here, the three lines used (UjuT, Uju.wMel and Uju.wt) contained over 99% identical nuclear background, following introgression with UjuT males, in order to control for any genetic differences between the lines that may have arisen through bottlenecking. The fecundity of the Uju.wMel line contrasts with the significant fecundity reduction previously observed for the *w*Pip transinfection of *Ae. albopictus* [35], and the hatch rate is much higher in the *w*Mel transinfected line than that previously observed for a *w*MelPop strain transinfection in *Ae. albopictus*, which averaged in the 10–20% range [13].

**Figure 2:**
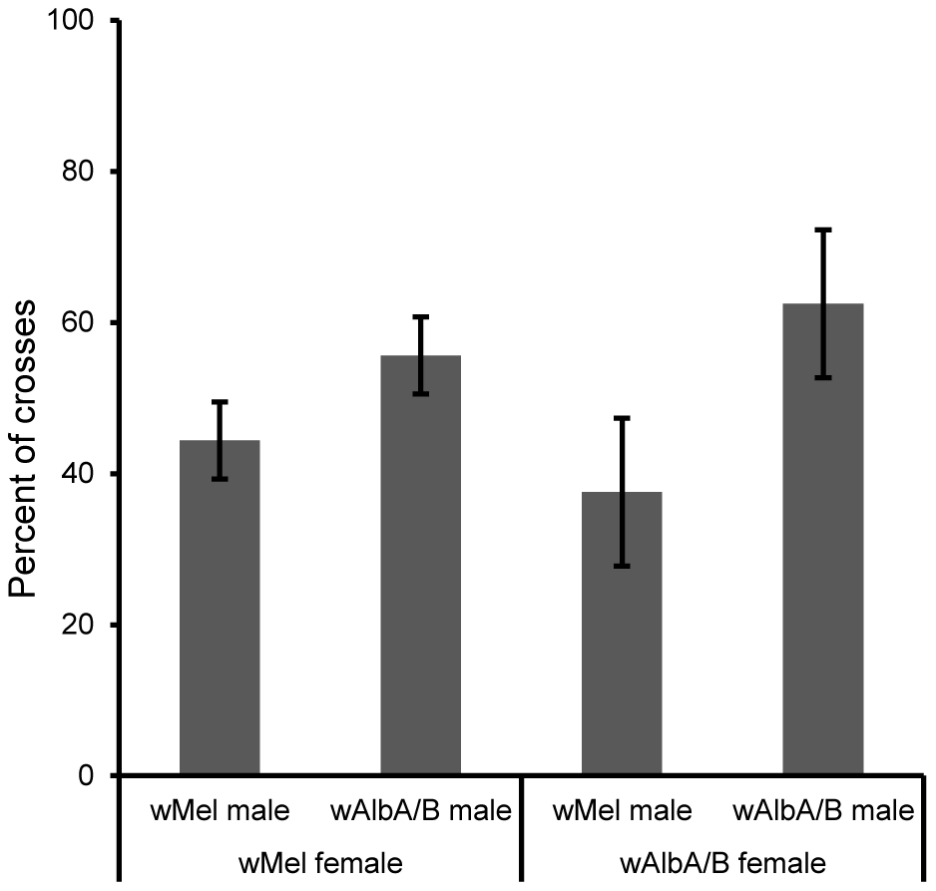
Longevity of Uju.wMel. The survival over time of three *Ae. albopictus* lines was assessed in two or three independent replicates of cages containing approximately 100 male and 100 female mosquitoes (total of 1600 mosquitoes in eight cages). A blood meal was provided at seven days, and again every 14 days. Dead mosquitoes were counted and removed every four days. Error bars show the SEM. Male Uju.wMel longevity was significantly increased compared to UjuT and Uju.wt.

Neither *w*Mel nor the natural *w*AlbA/B infections appear to have an effect on the longevity of female *Ae. albopictus* in these lab cage conditions (figure 2A). However, the longevity of Uju.wMel males was significantly increased compared to both the uninfected UjuT (*p* = 0, log rank test) and the naturally superinfected Uju.wt (*p* = 0, log rank test) (figure 2B). The average male lifespan was increased from 33.4 days (UjuT) or 33.6 days (Uju.wt) to 52.5 days (Uju.wMel). The reason for this increase in male lifespan is unknown. The lack of a significant difference in longevity in either sex between Uju.wt and UjuT contrasts with a previous report [9]. This difference between studies could have been a result of the seven generations of introgression that were used here to homogenize genetic backgrounds. No difference was found between the larval/pupal development times of Uju.wMel and Uju.wt; the mean time taken to eclose from egg hatch was 10.85±1.18 days (n = 505) and 10.87±1.52 days (n = 951) for Uju.wMel and Uju.wt respectively (p = 0.931 Log rank test; error represents one standard deviation).

**Figure 3:**
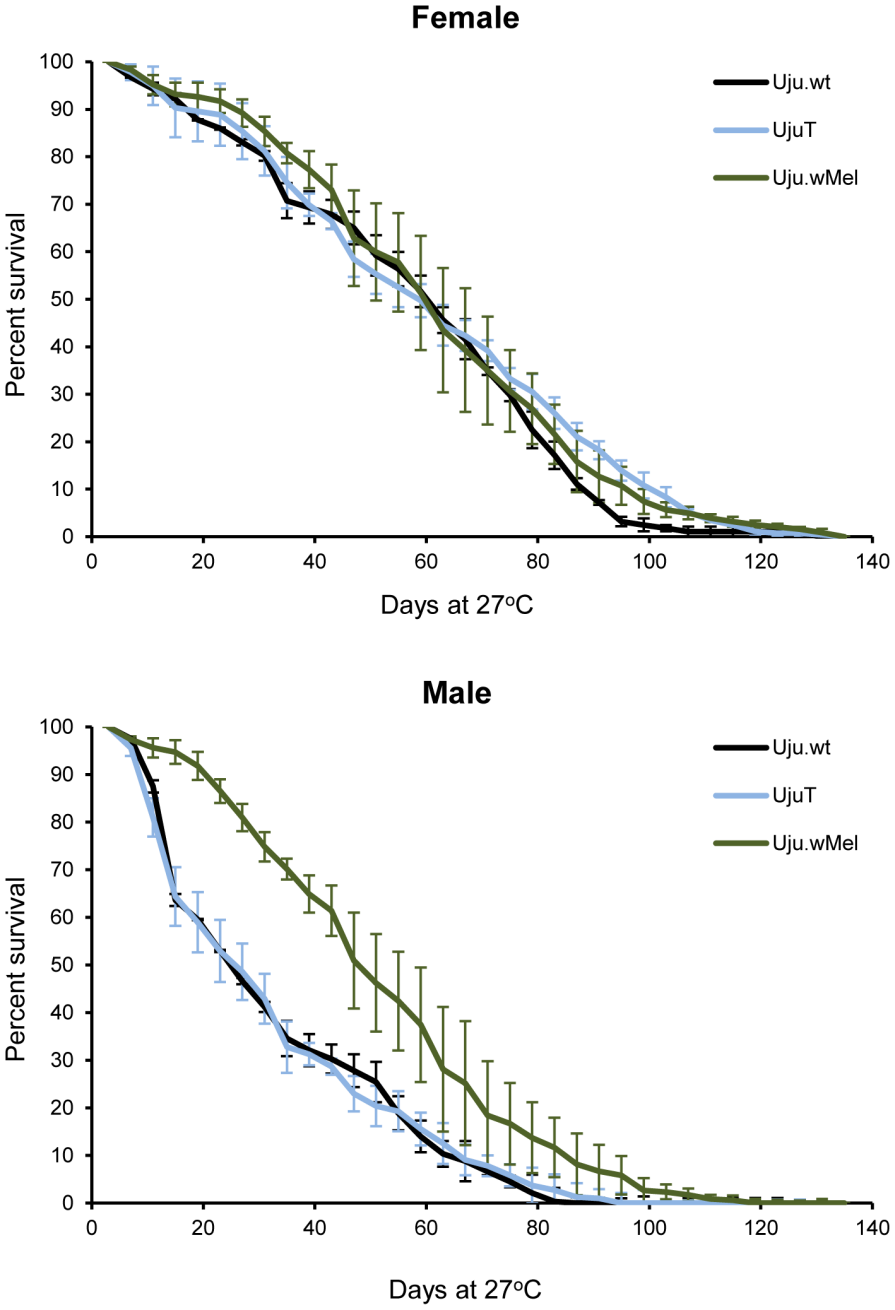
Mating competitiveness of Uju.wMel males. Competitiveness of Uju.wMel males was assessed using three independent replicates of 50 male Uju.wMel : 50 male Uju.wt (*w*AlbA/B) : 50 females of either Uju.wMel or Uju.wt (total of 300 females in six cages). Hatching embryos indicated a compatible cross where both male and female parents were infected with the same *Wolbachia*. Error bars show the SEM. No significant differences in male mating competitiveness were found between the two lines with Chi-squared analysis using a likelihood framework.

Male mating competitiveness of Uju.wMel males was compared to that of the naturally infected Uju.wt males using three independent replicates of 50 male Uju.wMel : 50 male Uju.wt : 50 females (either Uju.wMel or Uju.wt). Hatching eggs from an individual female indicated that a compatible mating had occurred with a male with the same *Wolbachia* strain, whilst no hatch indicated an incompatible cross (data from females with empty spermathecae were disregarded). No significant difference was found between the competitiveness of the wildtype and transinfected males under these conditions (*p* = 0.652, Chi-squared analysis using a likelihood framework) (figure 3).

### CHIKV susceptibility

**Figure 4:**
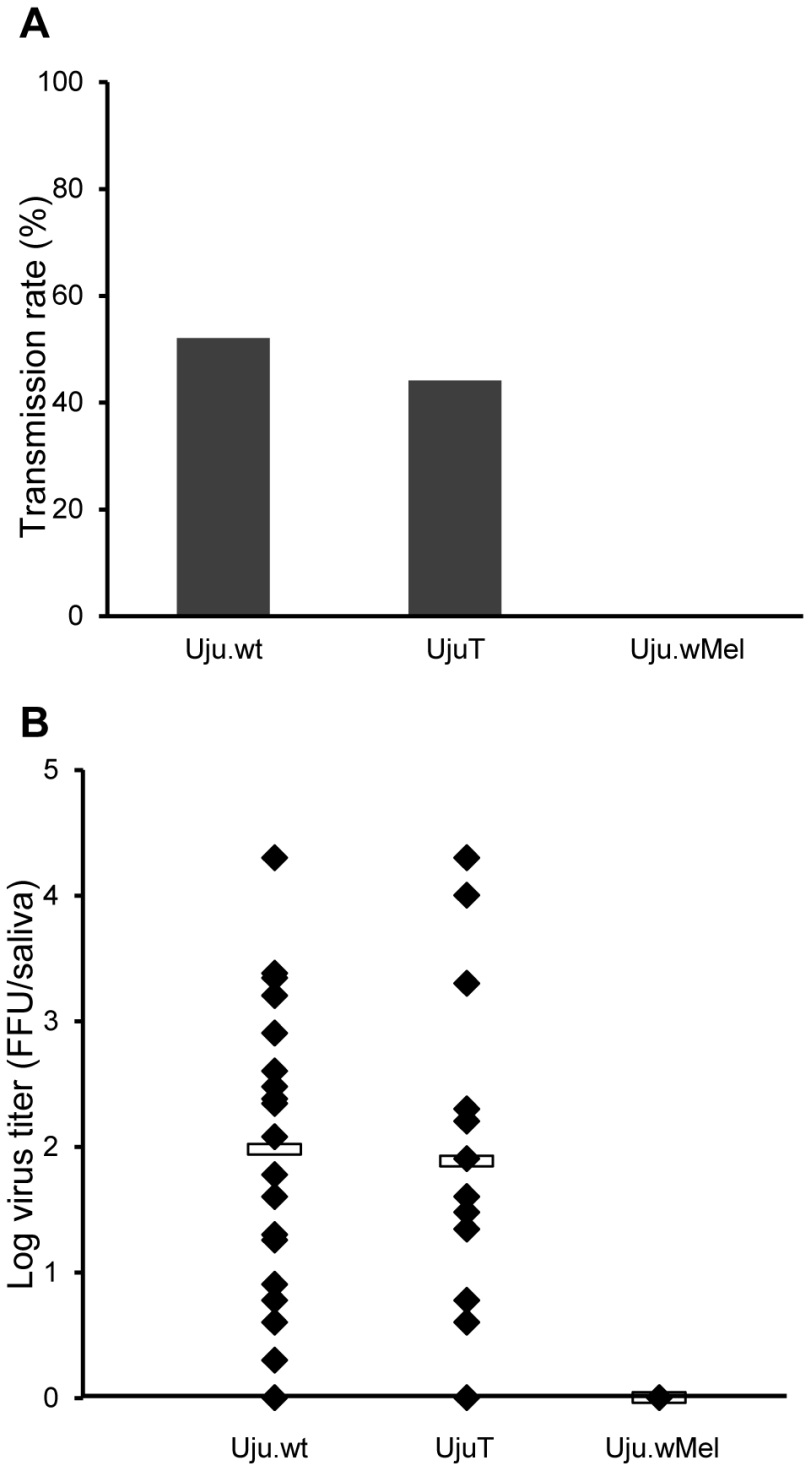
CHIKV challenge. Mosquitoes were allowed to feed on artificial blood meals containing virus suspension and 7 days post infection 35–50 females were used for forced salivation. Samples were titrated by focus fluorescent assay on *Ae. albopictus* C6/36 cells. The transmission rate was estimated as the percentage of mosquitoes with infectious saliva among tested mosquitoes (A). Saliva samples were titrated by focus fluorescent assay on C6/36 *Ae. albopictus* cell culture. The total number of plaques was counted and the titer was calculated as FFU/saliva (B). No significant difference was found between Uju.wt and UjuT viral titers using a Wilcoxon rank sum test.

The effect of *w*Mel on CHIKV inhibition was assessed by allowing adult females to feed on blood-meals containing a virus suspension, washed rabbit erythrocytes and 5 mM ATP. After 7 days, 35–50 females were anaesthetized on ice and salivation was induced. The saliva was titrated by focus fluorescent assay on *Ae. albopictus* C6/36 cells. CHIKV was present in 52% and 44% of Uju.wt and UjuT individuals' saliva, whilst no CHIKV was found in any Uju.wMel saliva (figure 4A), demonstrating complete viral inhibition caused by *w*Mel. No significant difference was observed between the viral titers found in Uju.wt and UjuT (*p* = 0.4129, Wilcoxon rank sum test) (figure 4B), suggesting that the presence/absence of *w*AlbA/B has no effect on CHIKV. This supports previous research showing no overall significant difference in CHIKV titers between *w*AlbA/B infected and uninfected *Ae. albopictus* [36].

## Discussion

In many regions of the world the most important disease transmission risk associated with *Ae. albopictus* is increasingly chikungunya [16], [17], and the relative importance of its transmission role compared to *Ae. aegypti* is probably greater for this disease than for dengue [18]–[23]. The data presented here provides a clear indication that the introduction to high frequency of the *w*Mel transinfection in wild populations of *Ae. albopictus* should prevent it from acting as a chikungunya vector in those populations, in addition to a likely impact on the transmission of dengue. The strong inhibition of CHIKV by *Ae. albopictus* is noteworthy in light of the fact that the viral strain used possesses the envelope glycoprotein E1-A226V mutation, which has greatly increased the transmission potential by *Ae. albopictus* [25]–[28]. It would be useful in future studies to conduct challenges with other viral strains, particularly one that carries the E2-L210Q mutation, although this does not produce as strong a positive effect on viral fitness in *Ae. albopictus* as the E1-A226V variant does [27]; thus the expectation is that the degree of inhibition of other CHIKV variants produced by *w*Mel would be at least as strong as that observed here. It was recently reported that the inhibition of CHIKV also occurs in the presence of *w*Mel transinfection in *Ae. aegypti* [37], and thus the use of *Wolbachia* offers considerable promise for future preventative control measures against this emerging disease.

The mechanisms of viral resistance conferred by *Wolbachia* have yet to be fully elucidated. It has been suggested that a reactive oxygen species (ROS)-dependent activation of the Toll pathway, and subsequent increased expression of antimicrobial peptides, is responsible or plays a major role in *w*AlbB-transinfected *Ae. aegypti* [38]. However, no evidence of increased antimicrobial peptide expression has been found in Uju.wMel [4]. These experiments included qRT-PCR of both a defensin and a cecropin, which were both highlighted as important effectors in *Ae. aegypti* infected with *w*AlbB [38]. Whilst the involvement of immune genes has not been ruled out, other possible pathogen inhibition mechanisms include direct production of ROS by *Wolbachia*, as observed for other bacteria [39], and competition for resources such as cholesterol between the host, the bacterium and the virus [7]. Interestingly, high experimental CHIKV titers have previously been shown to produce a reduction in density of wildtype *w*AlbA/B *Wolbachia* in *Ae. albopictus* [36], which supports the hypothesis of resource competition between them. It will be important, with respect to the effectiveness of disease control strategies, to examine whether 100% blockage of viral transmission by *w*Mel is maintained after many generations of carriage of this *Wolbachia* strain.

We previously reported that Uju.wMel is fully bidirectionally incompatible with a *w*AlbA/B wildtype line, which provides a mechanism for contained replacement of the naturally occurring wildtype *Wolbachia* strains in natural populations, since the two could not stably co-exist within a population as long as they are freely interbreeding, and the majority strain would replace the minority [4]. A mathematical model [15] provides an example of the introduction of *Wolbachia* into discrete seasonal populations of mosquitoes. It is clearly undesirable to release large numbers of biting females to achieve a population majority in a target population. However, by releasing a high percentage of males (the majority of adult males emerge before the females and pupae can also be separated into sexes by size) and beginning releases at the start of the rainy season, a relatively modest program of releases over a period of months should be sufficient to take a *Wolbachia* strain that produces complete bidirectional CI with wildtypes to fixation in a population if there are no major fitness costs of the transinfection under field conditions. At the same time there would be the benefit of suppressed overall population size of biting females due to the releases of incompatible males. Once established at 100% frequency, the transinfection would be resistant to moderate levels of immigration of wildtypes from surrounding populations, since immigrating wildtype females would be incompatible with a majority of the males they encountered.

The fitness parameters that have been measured here are all favorable with respect to the aim of introducing the *w*Mel transinfection into natural *Ae. albopictus* populations for the purpose of disease prevention. The *w*Mel-transinfected males were fully competitive with wildtype males in the simple cage mating assays employed. Extrapolations to what will occur under natural conditions must be made with caution. Semi-field contained greenhouse trials would give a better approximation to nature for longevity and mating competitiveness assays than insectary cages. Ultimately replacement dynamics can be fully assessed in open field trials; the relative effects on fitness of *w*Mel in the greater genetic heterogeneity of wild populations is another unknown. Inn general however, small-cage lab assays have been found to be a reasonably reliable indicator of field success in the *Aedes* release programs conducted to date [40], [41]. Detailed examinations of the relative effects of *Wolbachia* in *Ae. albopictus* on other life history parameters, particularly using different larval rearing conditions and examining effects on adult size, developmental time and larval and adult competitiveness, and also lifetime measures of fecundity, would also be useful future laboratory-based studies.

Even if significant reductions in the mating competitiveness of *w*Mel-infected males occurred under field conditions, this could be abrogated by a sufficiently high ratio of released males to wild type males. Various scenarios can be envisaged where the long-term effectiveness of such a *Wolbachia*-based strategy for disease control might gradually become compromised, such as the occurrence and spread of viral mutations that escape the inhibition, but such mutations are by no means inevitable. Based on all the data collected so far, the use of *w*Mel offers a promising new method to prevent or reduce dengue and chikungunya transmission by *Ae. albopictus*.
